# Optimal pre-plant irrigation and fertilization can improve biomass accumulation by maintaining the root and leaf productive capacity of cotton crop

**DOI:** 10.1038/s41598-017-17428-5

**Published:** 2017-12-07

**Authors:** Zongkui Chen, Hui Ma, Jun Xia, Fei Hou, Xiaojuan Shi, Xianzhe Hao, Abdul Hafeez, Huanyong Han, Honghai Luo

**Affiliations:** 1 Key Laboratory of Oasis Eco-Agriculture, Xinjiang Production and Construction Group, Shihezi University, 832003 Shihezi, Xinjiang China; 2 Cotton Physiology Lab for Efficient Production, College of Plant Science and Technology, Huazhong Agricultural University, 430070 Wuhan, Hubei China; 3 Cotton Institute, Xinjiang Academy Agricultural and Reclamation Science, 832003 Shihezi, Xinjiang China

## Abstract

Cotton is a major cash crop grown worldwide primarily for fiber and oil seed. As the most important cultural practices for cotton production, single pre-plant irrigation and basal fertilization for cotton plant growth and yield are well documented, but their coupling effects are poorly understood in arid regions. A 2-year outdoor pot trial was conducted to unravel the effects of pre-plant irrigation and basal fertilization on leaf area, root growth, biomass accumulation, and capacity of leaf area and root in cotton plant. Two pre-plant irrigations (i.e., W_80_, well-watered and W_0_, not watered) and two basal dressing fertilizations (F_10_, surface application and F_30_, deep application) were used in the experiments. The aboveground and reproductive biomass were highest in W_80_F_10_ after 69 days after emergence. Furthermore, W_80_F_10_ increased the root length in the 0–40 cm soil layer and the leaf area and improved the loading boll capacity of the effective root length and leaf area. The effective root length and leaf area had substantial direct effects on the aboveground and root biomass, respectively. Our data suggest that basal fertilizer surface application under adequate pre-plant irrigation is an effective strategy for optimal cotton production, which improves the coordination of water-nutrient absorption and photosynthetic areas and promotes assimilated distribution to the reproductive structures.

## Introduction

Cotton is the most significant fiber and commercial crop globally. The shortage of water resources and low water and nutrient availability have decreased cotton yields by 19 kg ha^−1^–750 kg ha^−1^ and 8–40%^1,2^. To obtain higher yields, farmers have increased the water input (the agricultural water input increased by 20% from 1965 to 2000)^3^ and agricultural nutrient application (the nitrogen rate for the highest yield was up to 300 kg ha^−1^ in Xinjiang, China, whereas the nitrogen rate for the highest cotton lint yield was 240 kg ha^−1^ in the Yangtze River Valley)^4^. These practices have resulted in numerous problems, including nutrient imbalance in the soil^5^, declining yields and quality^6,7^, and increasing soil salinization^8,9^. Therefore, it is important to studying the feasibility of optimal irrigation and fertilization without sacrificing the yield and fiber quality of cotton crop and to minimize the risk of environment pollution.

Different rates and modes of water and nutrient application can improve water-nutrient coordination to increase the effectiveness and productive capacity, which benefit biomass formation^10,11^. The physiological mechanism of the water-nutrient application rate and modes promotes biomass accumulation primarily by improving the photosynthetic capacity^12,13^, promoting the water-nutrient absorptive capacity of the roots^14,15^, and optimizing crop compensation^16,17^ and self-regulation^18^. Thus, the water-nutrient application rate and modes primarily increase the water-nutrient availability in the soil and the physiological activity of the leaves and roots to promote the photosynthetic capacity and the formation of dry matter. Notably, the water shortage has caused a lower nutrient use efficiency and increased the nutritional residues in the soil^10^. Several water-nutrient application modes exploit crop compensation and self-regulation capacities, which are beneficial for decreasing inputs but are not conducive to obtaining the highest yields^19^. Additionally, the water use efficiency is often considered to involve the ratio between physiological (i.e., transpiration and photosynthesis) and agronomic (i.e., yield and crop water use) measures^20^. In other words, the method by which irrigation is provided can affect the productive availability of a certain amount of water and nutrients, thereby affecting the water-nutrient efficiency. Thus, this study aimed to establish effective modes of water-nutrient application to increase coordination among the roots, the water and nutrients in the soil and the soil environment and to optimize self-regulation to obtain higher yields and a higher water-nutrient productive capacity.

Xinjiang is one of the major cotton growing regions in China, contributing 53.9% of the total national lint production^21^. However, the shortage of water resources and low water-nutrient availability have substantial impacts on cotton production. Numerous studies have shown that reasonable modes of water-nutrient management can obtain higher cotton yields^4,22^ while decreasing inputs and protecting the environment during agricultural production^23,24^. Commonly, no irrigation and fertilization are applied before the first flowering stage during the cotton management process in Xinjiang. The pre-plant irrigation and fertilization are generally considered the most important water and nutrient for promoting cotton plant growth^25,26^. However, limited information is available on their combined effects on water-nutrient absorptive area (root distribution), the photosynthetic area (leaf area), and biomass accumulation of cotton crop. This study aims to explore both single and interactive effects of pre-plant irrigation and fertilization on the cotton leaf area, root growth, biomass accumulation, and capacity of the leaf area and roots and to determine the quantitative relationships between these factors.

## Results

### Biomass accumulation and partitioning

**Figure 1:**
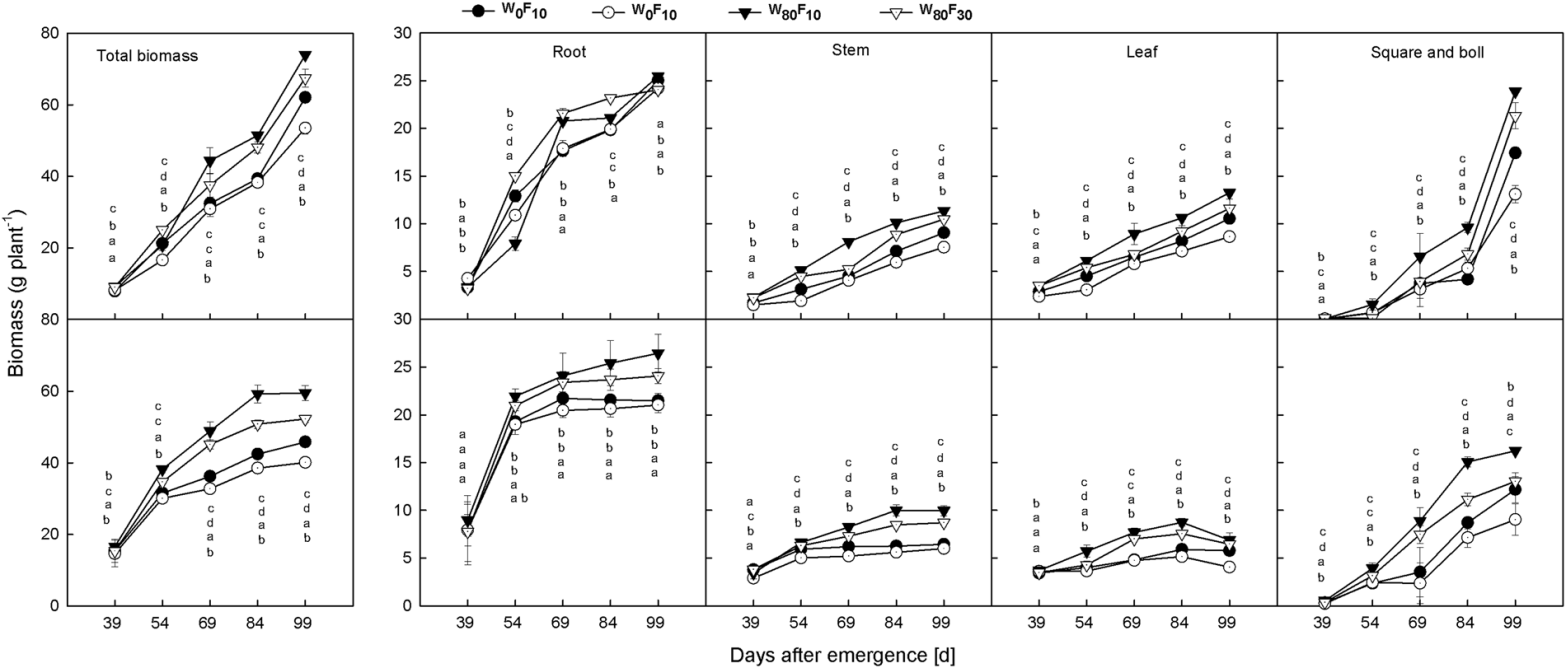
Change of the productive organ, leaf and stem and root biomass of cotton (g plant^−1^) at pre-plant watered (W_80_) or no watered (W_0_) and base fertilizer surface (F_10_) or deep (F_30_) application with the days after emergence in 2015 and 2016. Bars indicate SD (n = 3). The different letter within a column are significantly different (*P* < 0.05) according to Duncan’s multiple range test. From top to bottom, the letters within a column are marked following the order in W_0_F_10_, W_0_F_30_, W_80_F_10_ and W_80_F_30_.

Throughout the entire growth period, the accumulation of the stem and leaf biomass increased more after 54 days after emergence (DAE), whereas reproductive organs biomass increased rapidly after 84 DAE for all treatments (Fig. 1). In the W_80_ treatments, the leaf, stem, and reproductive organs and the total biomass were 29.7%, 31.6%, 48.0% and 22.4% greater than the corresponding values in the W_0_ treatments, respectively. The W_80_F_10_ treatment increased the leaf and stem biomass by 12.3% and 7.7%, respectively, and the reproductive organs and total biomass increased by 11.9% and 11.7%, respectively, compared to the measurements in the F_30_ treatment.

**Figure 2:**
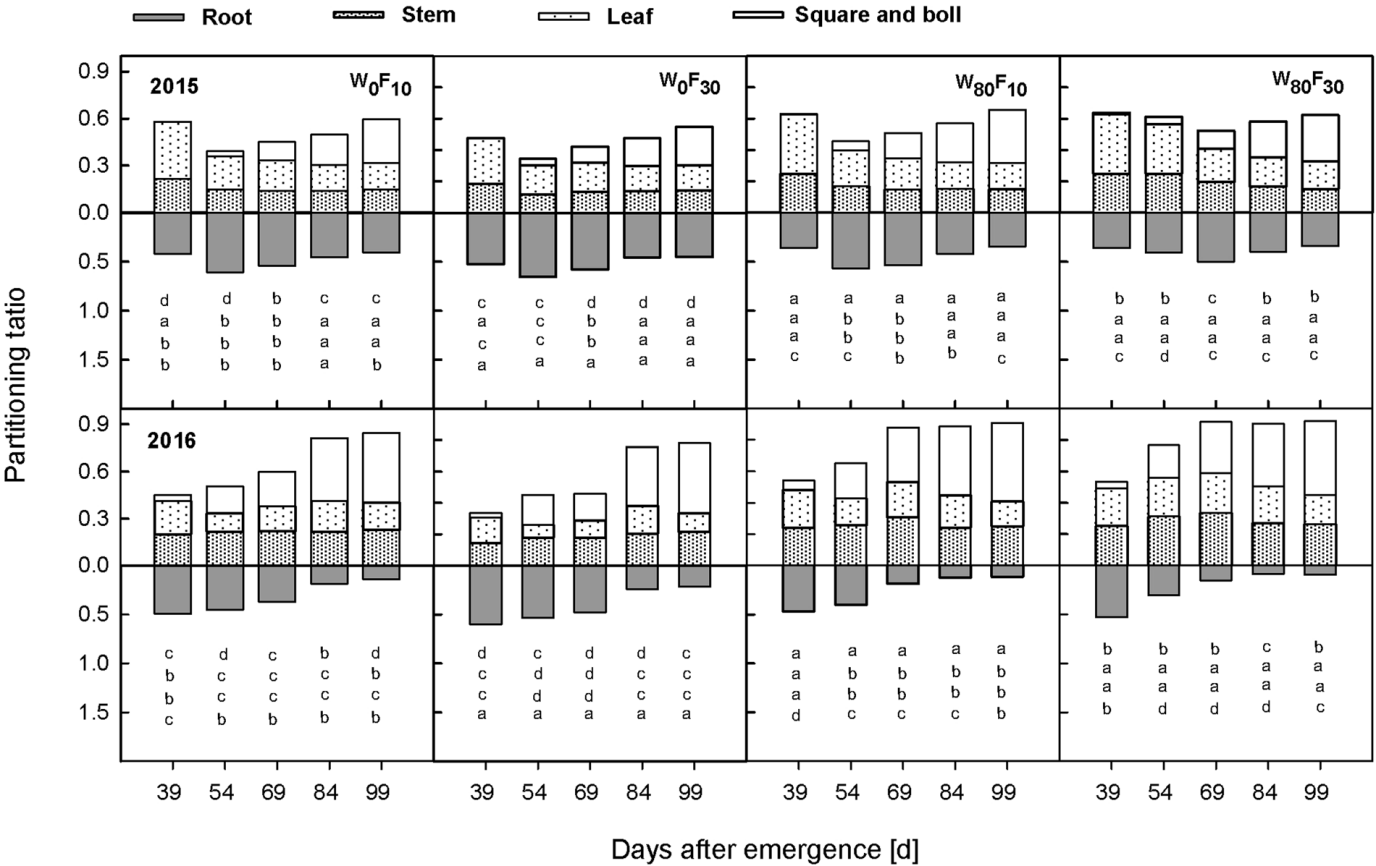
The change of the partitioning ratio in productive organ, leaf and stem and root partitioning ratio production of cotton at pre-plant watered (W_80_) or no watered (W_0_) and base fertilizer surface (F_10_) or deep (F_30_) application with the days after emergence in 2015 and 2016. The different letter within a line are significantly different (*P* < 0.05) according to Duncan’s multiple range test.

The root partitioning ratio was greater than the aboveground organ partitioning ratio before 69 DAE (Fig. 2). The stem and leaf partitioning ratio first increased and then slowly decreased, whereas the reproductive organ partitioning ratio rapidly increased during the entire growth stage. W_80_ decreased the root partitioning ratio by 8.3–41.1% compared to the ratio in W_0_, whereas the stem, leaf and reproductive organ partitioning ratios in the W_80_ treatments were significantly increased by 16.1–44.1%, 17.1–75.2% and 7.9–2.9%, respectively, compared to the ratios observed for W_0_. In W_0_, F_10_ decreased the root partitioning ratio by 15.4–22.1% and increased the stem, leaf and reproductive organ partitioning ratios by 5.7–40.1%, 10.8–48.5% and 10.0–29.8%, respectively, compared with the ratios observed for the F_30_ treatment. Under the W_80_ condition, the F_10_ treatment resulted in 11.2–20.1%, 5.2–11.8% and 9.1–14.9% decreases in the root, stem and leaf partitioning ratios, respectively, and 6.4–39.9% greater reproductive organ partitioning compared to the values obtained with the F_30_ treatment.

### Changes in leaf area and effective root growth

**Figure 3:**
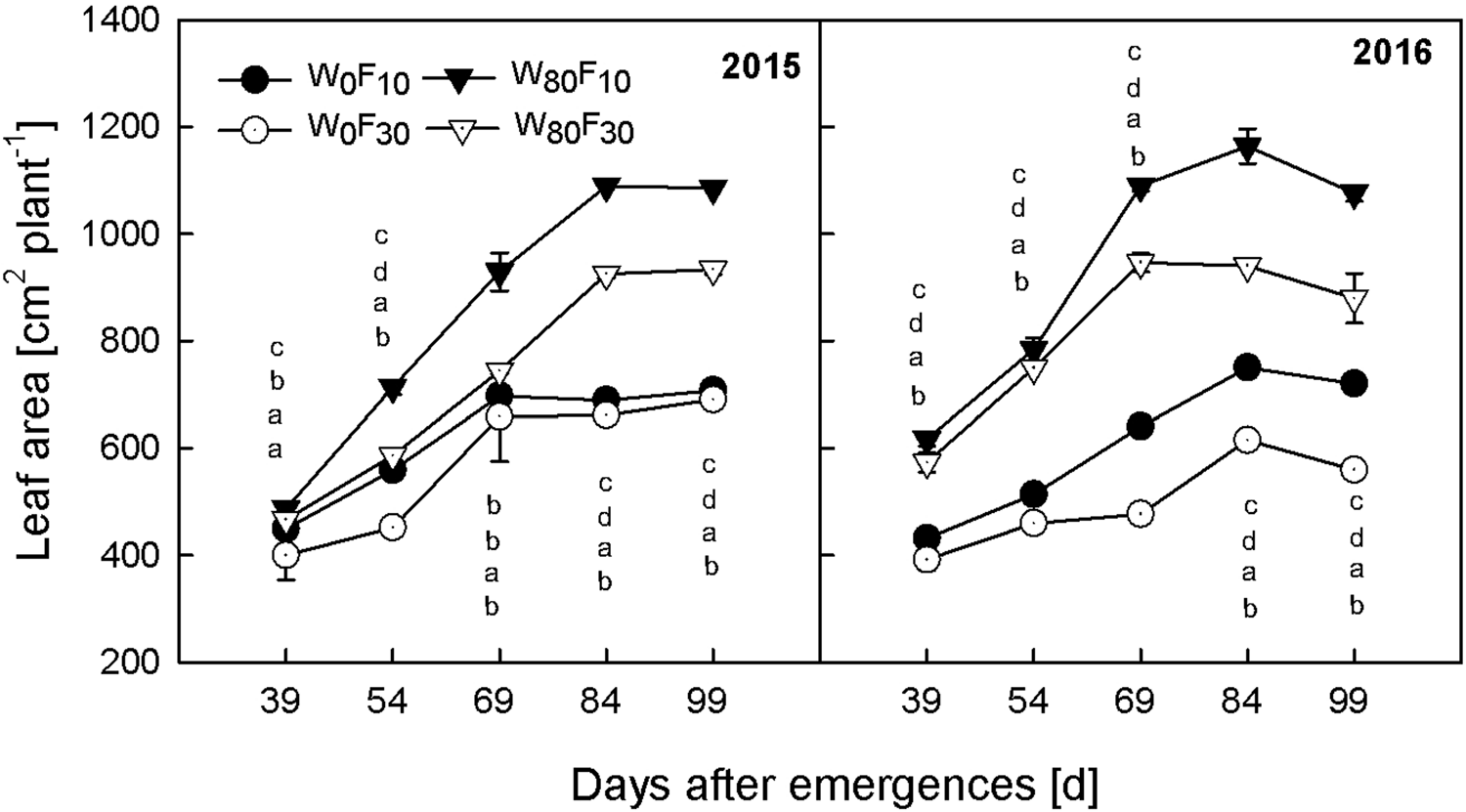
Change in the leaf area (cm^−2^) of cotton at pre-plant watered (W_80_) or no watered (W_0_) and base fertilizer surface (F_10_) or deep (F_30_) application with the days after emergence in 2015 and 2016. Bars indicate SD (n = 3). The different letter within a column are significantly different (*P* < 0.05) according to Duncan’s multiple range test.

The leaf area increased with the DAE (Fig. 3). All treatments exhibited similar response patterns during the growth period. Compared to W_0_, W_80_ resulted in a 12.2–49.2% higher leaf area. Maximum values were observed at 84 DAE under the W_80_ condition compared to the values obtained at 69 DAE under the W_0_ condition. The leaf area in F_10_ was significantly higher than the leaf area in F_30_. Among all treatments, the highest leaf area was recorded in W_80_F_10_, and the value of the leaf area was 4.3–24.9% greater than the value in W_80_F_30_.

**Figure 4:**
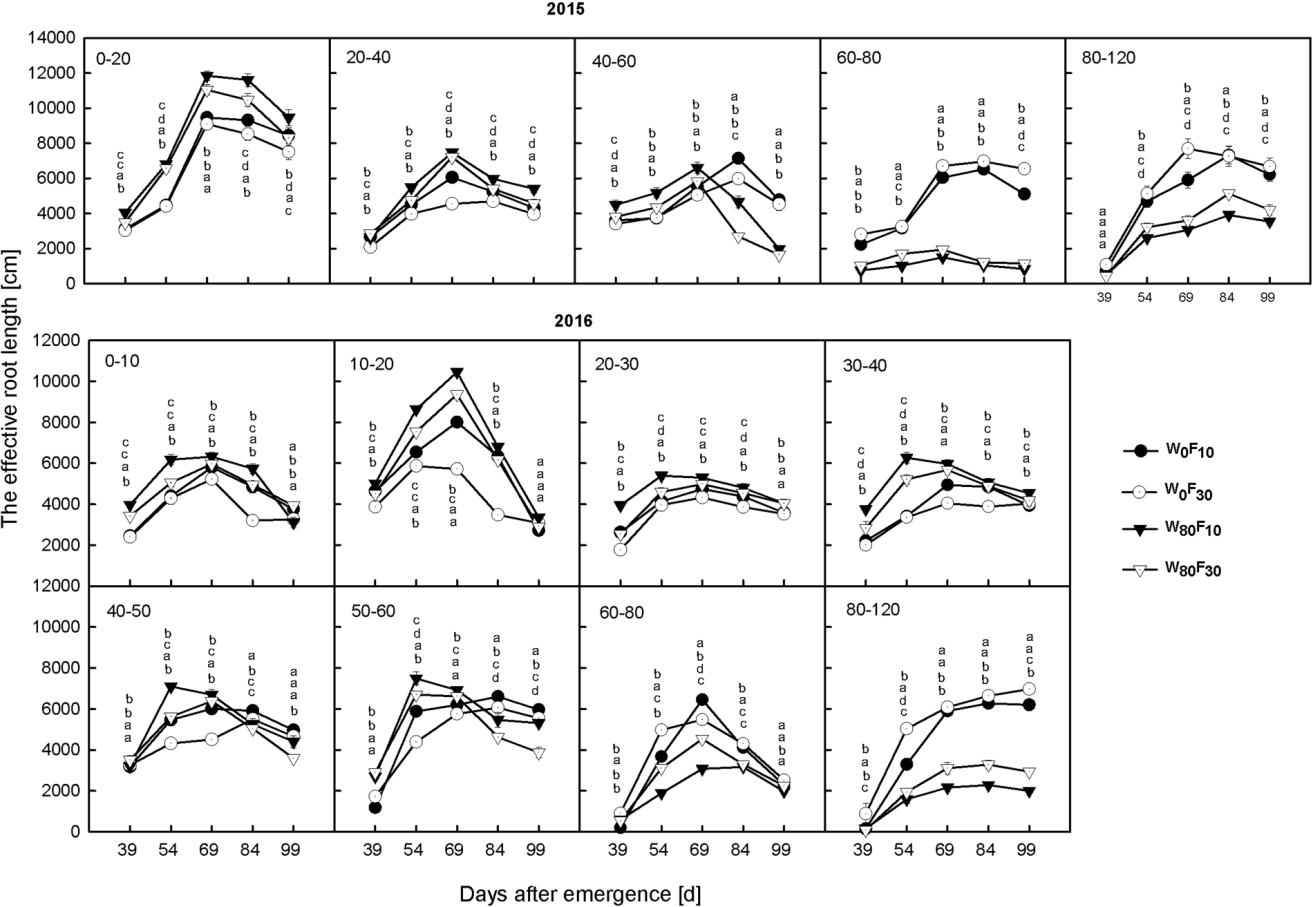
Changes in the effective root length in different vertical soil layer (from 0 cm to 120 cm soil layer) at pre-plant watered (W_80_) or no watered (W_0_) and base fertilizer surface (F_10_) or deep (F_30_) application with the days after emergence in 2015 and 2016. Bars indicate SD (n = 3). The different letter within a column are significantly different (*P* < 0.05) according to Duncan’s multiple range test.

The effective root length first increased and then decreased in the entire soil layer during the entire growth period (Fig. 4). Compared to W_0_, W_80_ resulted in 11.3–50.7% higher effective root lengths in the 0 cm–40 cm soil layer during the entire growth stage and 38.1–73.1% lower values in the 40 cm–60 cm soil layer after 69 DAE. Under the W_80_ condition, the effective root length in F_10_ increased by 9.6–42.3% in the 0 cm–60 cm soil layer but had a 16.0–65.7% lower value in the 80 cm–120 cm soil layer than in the F_30_ treatment during the entire growth stage.

### Relationship of the root distribution and leaf area with the biomass accumulation and partition

The effective root length had a significant positive (*P* < *0.01*) relationship with the leaf area, root biomass, vegetative organ and reproductive organ biomass (Table 1). The leaf area also had a significant positive relationship (*P* < *0.01*) with the root biomass, vegetative organ and reproductive organ biomass. The vegetative organ biomass had a significant positive (*P* < *0.01*) relationship with the reproductive organ biomass.

**Table 1:** Relationship (n = 120) among the effective root length (cm), leaf area (cm^−2^), root biomass (g plant^−1^), vegetative organ biomass (g plant^−1^) and reproductive organ biomass (g plant^−1^) at pre-plant watered (W_80_) or no watered (W_0_) and base fertilizer surface (F_10_) or deep (F_30_) application with 2015 and 2016.

	The effective root length (cm)	Leaf area (cm^2^)	Root biomass (g plant^−1^)	vegetative organ biomas (g plant^−1^)	Reproductive organ biomss (g plant^−1^)
The effective root length (cm)	1.00	0.473**	0.597**	0.452**	0.290**
Leaf area (cm^2^)	0.000	1.00	0.381**	0.706**	0.609**
Root biomass (g plant^−1^)	0.000	0.000	1.00	0.819**	0.757**
Vegetative organ biomass (g plant^−1^)	0.000	0.000	0.000	1.00	0.906**
Reproductive organ biomass (g plant^−1^)	0.001	0.000	0.000	0.000	1.00

* and ** means significantly different (*P* < 0.05, *P* < 0.01) according to Duncan’s range test.

The path analysis (Table 2) also showed that the effective root length, leaf area and vegetative organ dry matter had larger direct effects on the root biomass accumulation, whereas the effective root length and leaf area had larger indirect effects on the root biomass through vegetative organ biomass accumulation. Vegetative organ and reproductive organ biomass had negative effects on the root biomass accumulation. The leaf area and root biomass had direct effects on the vegetative organ biomass. Additionally, the vegetative organs had a competitive effect with the reproductive organs on root biomass accumulation. The vegetative organs had a larger direct effect on reproductive organ biomass accumulation, whereas the effective root length, leaf area and vegetative organs had larger indirect effects on reproductive organ biomass accumulation.

**Table 2:** Path analysis (n = 120) for the direct or indirect effect on dry matter accumulation by the effective root length (cm), leaf area (cm^−2^), root dry matter (g plant^−1^), vegetative organ dry matter (g plant^−1^) and reproductive organ dry matter (g plant^−1^) at pre-plant watered (W_80_) or no watered (W_0_) and base fertilizer surface (F_10_) or deep (F_30_) application with 2015 and 2016.

	Root biomass (Y1)				Vegetable organs biomass (Y2)				Reproductive organs biomass (Y3)			
	X1	X2	Y2	Y3	X1	X2	Y1	Y3	X1	X2	Y1	Y2
The effective root length (X1)	0.4296	−0.2455	0.3195	0.093	−0.0654	0.1678	0.2417	0.117	−0.2738	0.0575	0.1905	0.3167
Leaf area (X2)	0.2033	0.5187	0.5015	0.1953	−0.0309	0.3547	0.1545	−0.2456	−0.1296	0.1216	0.1218	0.4971
Root biomass (Y1)					−0.039	0.1353	0.4051	−0.3036	−0.1634	0.0464	0.3193	0.5528
Vegetable organs biomass (Y2)	0.1981	−0.3755	0.6929	−0.2894					−0.1263	0.088	0.057	0.6868
Reproductive organs biomass (Y3)	0.125	−0.3169	−0.4275	−0.3196	−0.019	0.2167	0.03059	0.0402				

### Biomass production capacity of the leaf area and roots and ratio of the effective root length to leaf area

**Figure 5:**
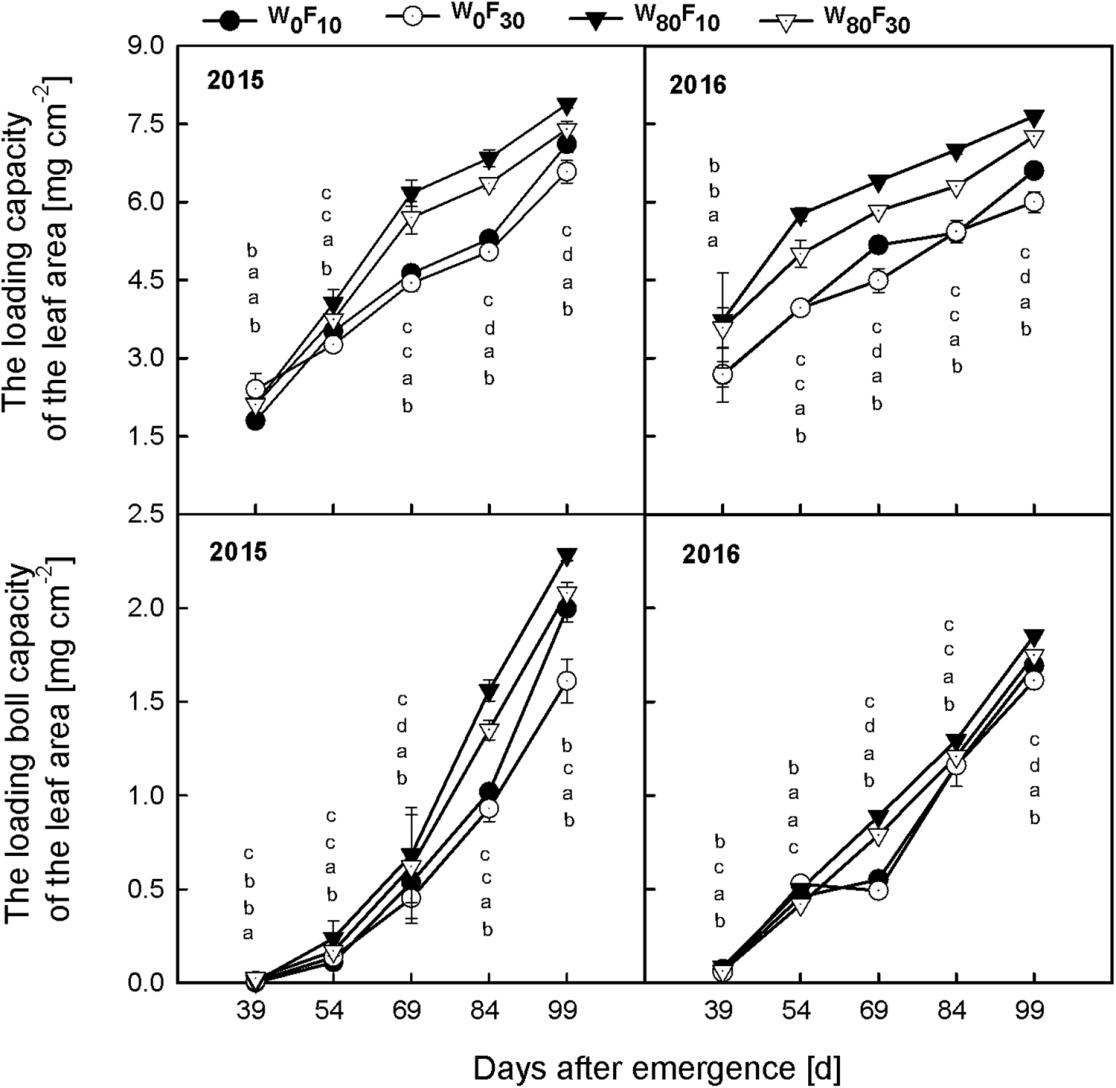
Change in the loading capacity (**A** and **B**) or loading boll capacity (**C** and **D**) of leaf area of cotton (mg cm^−2^) at pre-plant watered (W_80_) or no watered (W_0_) and base fertilizer surface (F_10_) or deep (F_30_) application with the days after emergence in 2015 and 2016. Bars indicate SD (n = 3). The different letter within a column are significantly different (*P* < 0.05) according to Duncan’s multiple range test.

The loading capacity (Fig. 5A,B) and the loading boll capacity (Fig. 5C,D) of the leaf area exhibited a rapidly increasing trend over the entire growth stage. The loading capacity and loading boll capacity of the leaf area in W_80_ were 0.9–30.9% and 21.0–65.9% greater than the capacities in the W_0_ treatment after 54 DAE. In the W_80_ treatment, F_10_ resulted in a 1.2–8.5% and a 9.8–40.2% higher loading capacity and boll loading capacity, respectively, than the F_30_ treatment after 54 DAE.

**Figure 6:**
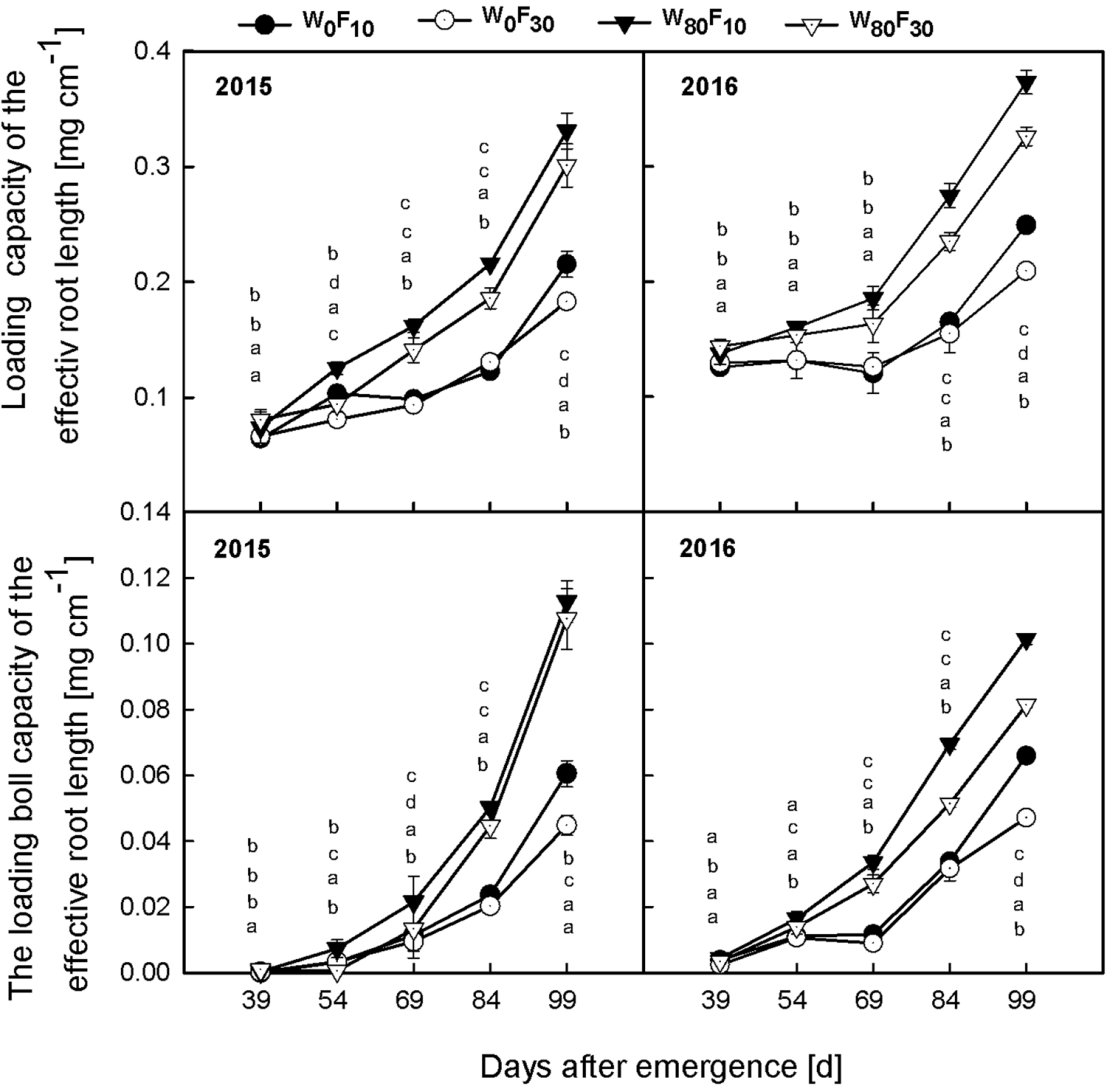
Change in the loading capacity (**A** and **B**) or loading boll capacity (**C** and **D**) of the effective root length (mg cm^−1^) of cotton at pre-plant watered (W_80_) or no watered (W_0_) and base fertilizer surface (F_10_) or deep (F_30_) application with the days after emergence in 2015 and 2016. Bars indicate SD (n = 3). The different letter within a column are significantly different (*P* < 0.05) according to Duncan’s multiple range test.

The loading capacity (Fig. 6A,B) and boll loading capacity (Fig. 6C,D) of the effective root length gradually increased before 69 DAE and then rapidly increased after 69 DAE. The loading capacity and boll loading capacity of the effective root length were 5.6–27.9% and 16.3–35.1% greater for the W_80_ treatment than for the W_0_ treatment, respectively. Under the W_80_ condition, the loading capacity and boll loading capacity of the effective root length were greater by 10.0–33.1% and 4.9–61.7%, respectively, in the F_10_ than in the F_30_ treatment.

**Figure 7:**
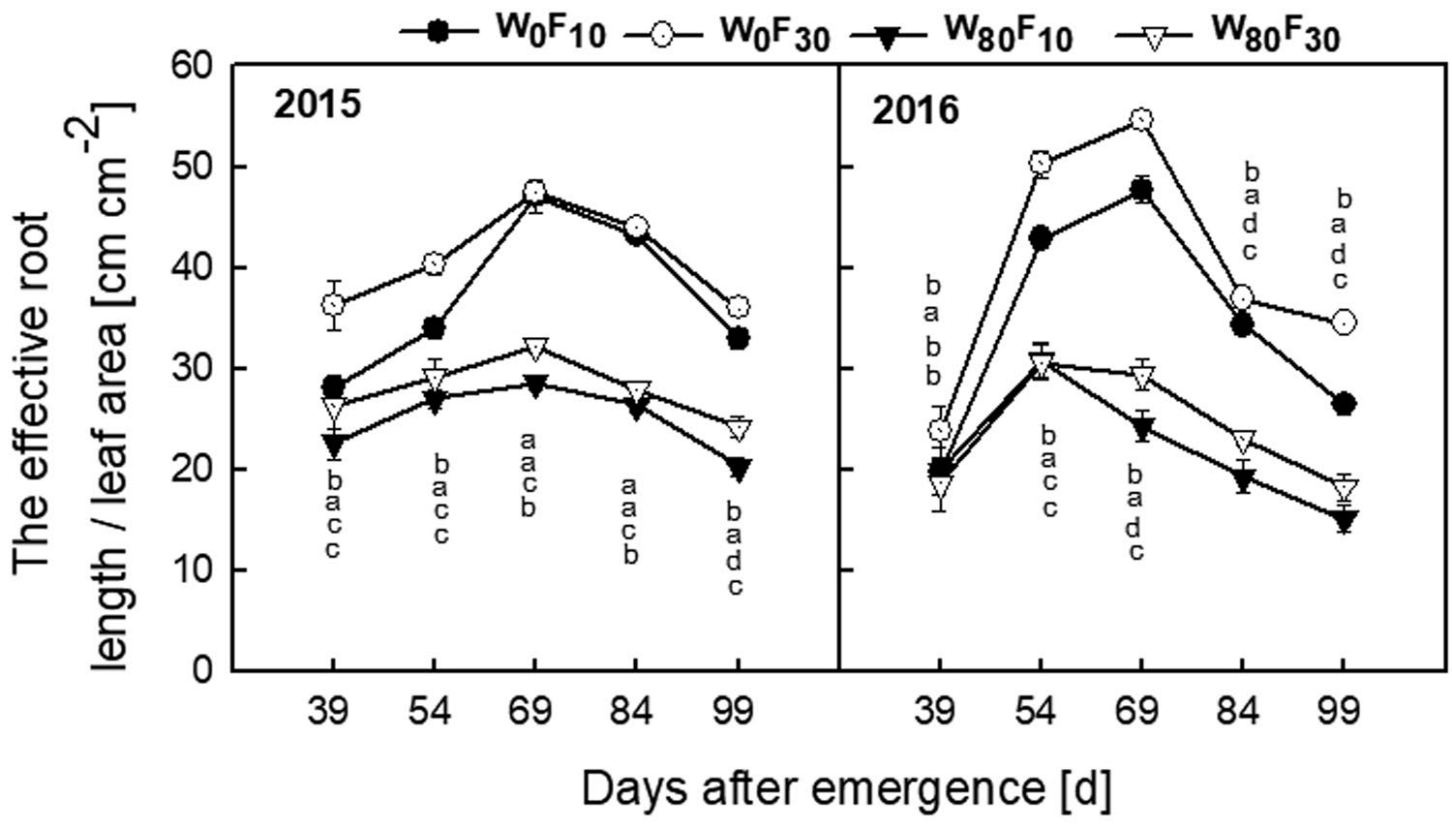
Change in the ratio of the relative root length to leaf area (cm^1^ cm^−2^) of cotton at pre-plant watered (W_80_) or no watered (W_0_) and base fertilizer surface (F_10_) or deep (F_30_) application with the days after emergence in 2015 and 2016. Bars indicate SD (n = 3). The different letter within a column are significantly different (*P* < 0.05) according to Duncan’s multiple range test.

The ratio of the effective root length to the leaf area (Fig. 7) first increased and then decreased, with the peak appearing at 69 DAE. The value in the W_80_ treatment was significantly lower by 31.4–60.3% than the value in the W_0_ treatment. Under the W_80_ condition, F_10_ was lower by 6.2–19.4% than the value under the F_30_ treatment during the entire growth stage.

### Relative growth rate of the leaf area and the effective root length

**Figure 8:**
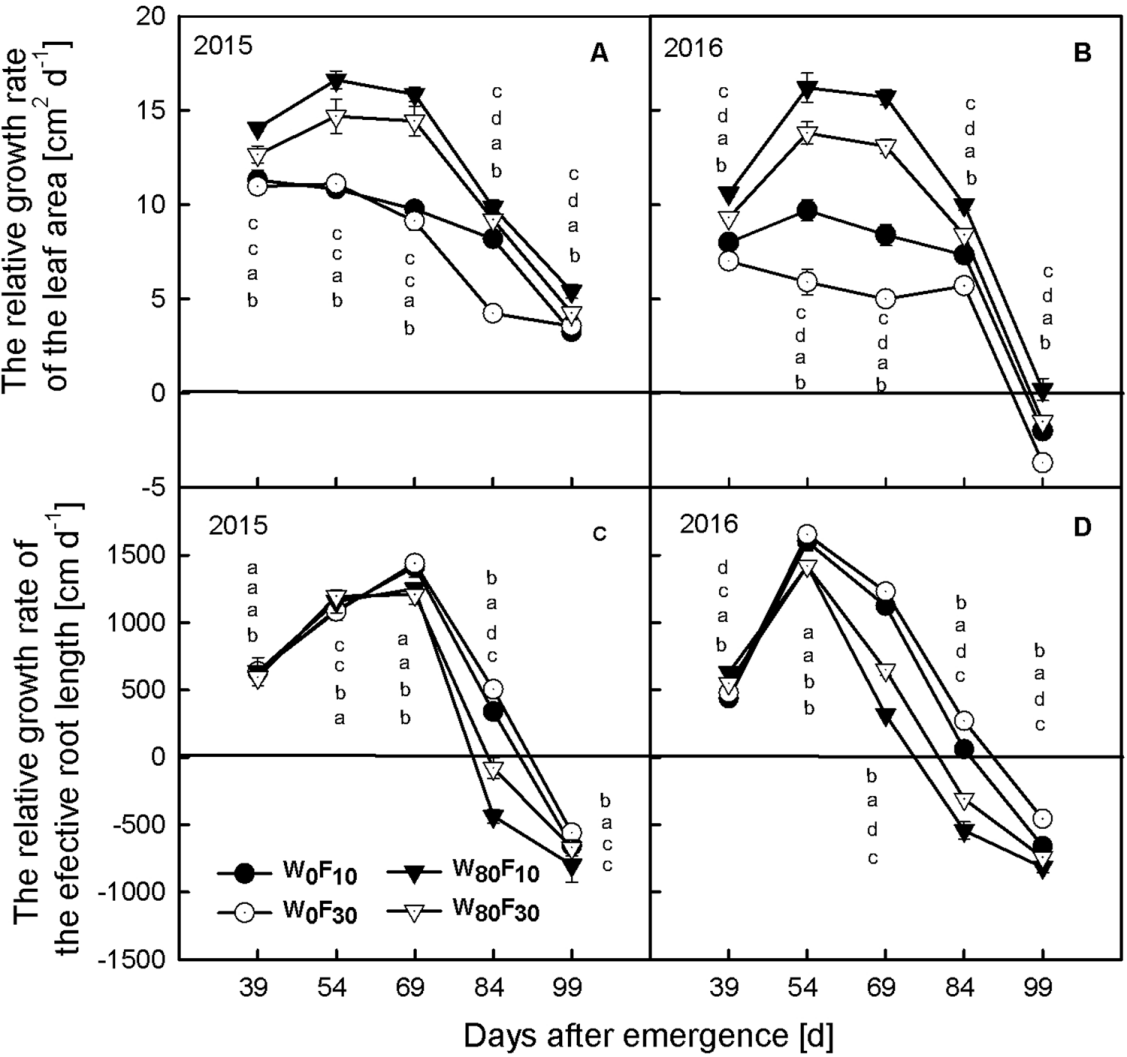
Change in the relative growth rate of leaf area (**A** and **B**, cm^2^ d^−1^) and the total effective root length (**C** and **D**, cm d^−1^) of cotton at pre-plant watered (W_80_) or no watered (W_0_) and base fertilizer surface (F_10_) or deep (F_30_) application with the days after emergence in 2015 and 2016. Bars indicate SD (n = 3). The different letter within a line are significantly different (*P* < 0.05) according to Duncan’s multiple range test.

The relative growth rate of the leaf area (Fig. 8A,B) and the total effective root length (Fig. 8C,D) were higher before 69 DAE and began to rapidly decrease after 69 DAE.

The relative growth rate of the leaf area in the W_80_ treatment was higher by 32.7–92.3% than in the W_0_ treatment during the entire growth stage. Under the W_80_ condition, the F_10_ treatment resulted in a 14.0–19.9% greater leaf area relative growth rate than under the F_30_ treatment.

The total effective root length relative growth rate was significantly lower (*P* < *0.05*) in the W_80_ treatment than in the W_0_ treatment after 54 DAE. Under the W_80_ condition, the effective root length relative growth was significantly higher (*P* > *0.05*) in the F_10_ treatment than in the F_30_ treatment after 69 DAE. Additionally, after 84 DAE, the effective root length relative growth rate in F_10_ decreased relatively slowly compared with the growth rate in the F_30_ treatment.

**Figure 9:**
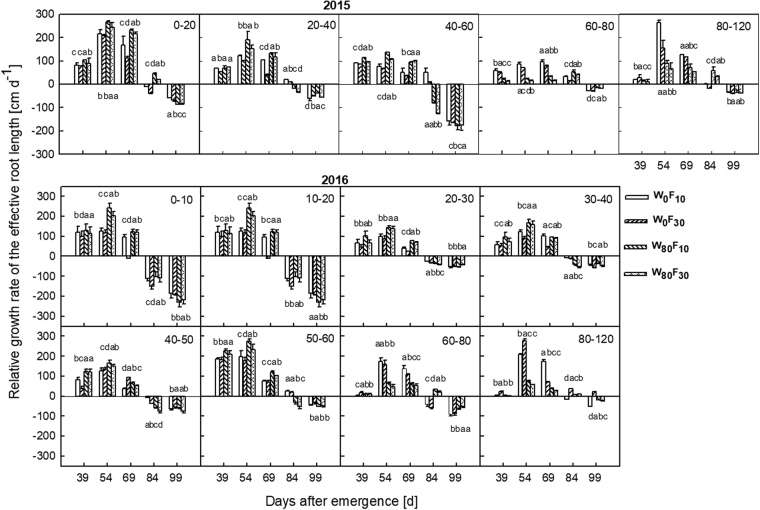
Changes in the relative growth rate of the effective root length in different vertical soil layer (from 0 to 120 cm soil layer) at pre-plant watered (W_80_) or no watered (W_0_) and base fertilizer surface (F_10_) or deep (F_30_) application with the days after emergence in 2015 and 2016. Bars indicate SD (n = 3). The different letter within a line are significantly different (*P* < 0.05) according to Duncan’s multiple range test.

Compared to the W_0_ treatment, the relative growth rate of the effective root length (Fig. 9) in the W_80_ treatment was greater by 20.5–76.5% in the 0 cm–40 cm soil layer and lower by 14.1–69.5% in the 60 cm–120 cm soil layer before 69 DAE. Additionally, W_80_ continued to exhibit 15.5–35.6% and 14.3–19.6% higher relative growth rates for the effective root length in the 0 cm–20 cm and 60 cm–120 cm soil layers, respectively, with 9.6–42.3% lower values in the 20 cm–60 cm soil layer than the values obtained with W_0_ after 69 DAE. Under the W_80_ condition, F_10_ resulted in a 7.0–46.7% higher relative growth rate of the effective root length in the entire soil layer before 69 DAE and 4.7–34.1% lower values in the 0 cm–120 cm soil layer after 69 DAE than F_30_.

## Discussion

Crop yield is closely related to biomass accumulation and partitioning, and increasing reproductive organs biomass are important for optimizing the crop yield^27,28^. In the present study, the W_80_F_10_ treatment exhibited the highest aboveground and reproductive organ biomass. Consequently, we suggest that coupling adequate pre-plant irrigation and basal fertilizer surface application as a high-yield and high-efficiency cultivation technique in the arid areas of Xinjiang China.

How does coupling the pre-planting irrigation and basal fertilizer application increase the aboveground biomass accumulation and partitioning? Research has shown that biomass accumulation reflects the source size^29^, whereas the partitioning ratio reflects the sink strength^30,31^. Therefore, we assumed that one important reason for the larger source capacity of the vegetative organs and the largest sink strengths of the reproductive organs at 69 DAE under W_80_F_10_ was the low availability of sources for the roots during the entire growth period. Greater vegetative organs biomass can easily increase the source size of these organs and transform the source into a sink because the source and the sink represent a change concept in different growth stages, particularly in woody or perennial plants^32–34^. Possible explanations include root redundancy or greater dry matter partitioning in roots under deficit conditions, such as water and nutrient deficits. However, for crops, water-nutrient support and protection largely ameliorate extreme conditions^35^, which ensures that the basic needs of crops to water-nutrient avoid excessive competition between crops. Thus, the W_80_F_10_ treatment supported the available source size of the roots and promoted the greatest source size of the vegetative organs, whereas the sink strength of the reproductive organs promoted an increase in the biomass accumulation in the aerial plant parts and partitioning to the reproductive organs.

How do the source and sink regulate the accumulation and partitioning of biomass? The leaf represents the primary photosynthetic organ^15^, and the root represents the primary means of water-nutrient absorption^36^. Thus, the source sizes of the root and vegetative organs in the aerial plant parts were loaded by an effective root distribution (i.e., the effective root length)^37,38^ and effective photosynthetic area (i.e., the leaf area)^39,40^, which is consistent with our results. Additionally, the path analysis results showed that the effective root length primarily increased the source size of the roots, whereas the leaf area primarily extended the source size of the aerial vegetative organs. Moreover, the sources of the aerial vegetative organs directly supplied the sink of the reproductive organs, whereas the roots were the indirect supply to the sink of the reproductive organs through the sources of the aerial vegetative organs. Numerous studies have shown that the development of sinks in crop reproductive organs is directly related to the source size of the aerial vegetative organs^28^ and leaf area^41^, whereas root biomass was supported by the aerial parts of the plant during the early growth period^34^. The effective root distribution (during the entire growth stage) promoted water-nutrient absorption, which supported dry matter accumulation in the plant^30^. Therefore, the effective root length and leaf area primarily increased the source size (consisting of the roots and vegetative organs in the aerial parts of the plant) after 69 DAE, which directly supported the sink of the reproductive organs.

How do an effective root length and leaf area affect the sink and source sizes? The W_80_F_10_ treatment had the highest productive capacity with respect to the root length and leaf area, the highest relative growth rate of the leaf area before 69 DAE, and a lower reduction in the leaf area after 69 DAE. Additionally, W_80_F_10_ had a slower relative growth rate of the effective root length and a lower ratio of the effective root length to the leaf area during the entire growth stage. These results indicated that improving the productive ability of the root and leaf area and the leaf area relative growth rate as well as coordinating the effective root length and leaf area played important roles in promoting the larger source sizes of the aerial vegetative organs and the sinks of the reproductive organs. One possible explanation is that reasonable water-nutrient application increased the available water and nutrients in the soil, which increased the effective root length^42^ and its relative growth rate^43^ to obtain adequate water and nutrients. This process was beneficial for extending the leaf area^44^ and promoting biomass formation^4^. Additionally, the leaf area was the main locus of gas exchange^15^, and the effective root length was the main organ for water-nutrient absorption^36^. Therefore, reasonable water-nutrient application increased the available water and nutrients in the soil, causing a relatively lower effective distribution and higher leaf area and lowering the ratio of the effective root length to leaf area, which coordinated the root distribution, leaf gas change and water and nutrients in the soil.

The roots and leaf areas of cotton should have specific functional periods and key soil layers with respect to the root distribution. How are the functional period or key soil layer with respect to the root distribution affected by spatial water-nutrient application? In this study, W_80_F_10_ increased the effective root length (diameter less than 0.5 mm) in the 0 cm–40 cm soil layer and decreased the effective root length in the 60 cm–120 cm soil layer (forthcoming). These outcomes were beneficial for intercepting the water and nutrients (following irrigation water application and in the soil) in the surface soil layer, and the less effective roots below the 60 cm soil layer obtained adequate moisture in the deep layer^25,44^ through root absorption and the utilizable water and nutrients in the soil. In turn, more photosynthates were formed due to a higher leaf gas exchange, the photosynthetic effect was promoted, and the increased photosynthates provided the material basis for root growth^45^. Additionally, due to the increasing utilizable water and nutrients, root development primarily occurred before the full flowering stage^46^. Irrigation water application promoted effective root distribution and increased the growth rate of the effective root length in the 0 cm–40 cm soil layer^47^. Numerous studies^43,44^ have shown that reasonable water-nutrient application increases the utilizable water and nutrients, thereby increasing the partitioning of biomass to the leaves and promoting the photosynthetic area. When nutrients were applied in the 30 cm–40 cm soil layer in the W_80_F_30_ treatment, the nutrients moved down to the deep layer (below 40 cm) following irrigation. As a result, the effective root length above 40 cm decreased, and the absorption of nutrients following irrigation decreased.

## Conclusion

The W_80_F_10_ treatment promoted the effective root length and its relative growth rate in the 0–40 cm soil layer and decreased these variables in the 60 cm–120 cm soil layer. An increasing leaf area and its relative growth rate were observed over the entire growth stage. This process improved the productive ability of the roots and leaves and the coordinative ability of the effective root length and leaf area. This effect increased the resource size of the roots and the aerial vegetative organs and the sink strength of the vegetative organs, which promoted biomass accumulation in the aerial plant parts and partitioning to the reproductive organs after the flowering stage. Thus, coupling of the basal fertilizer surface application and adequate pre-plant irrigation is an effective strategy for optimal cotton production for agriculture productivity in arid areas.

## Materials and Methods

### Site description

**Figure 10:**
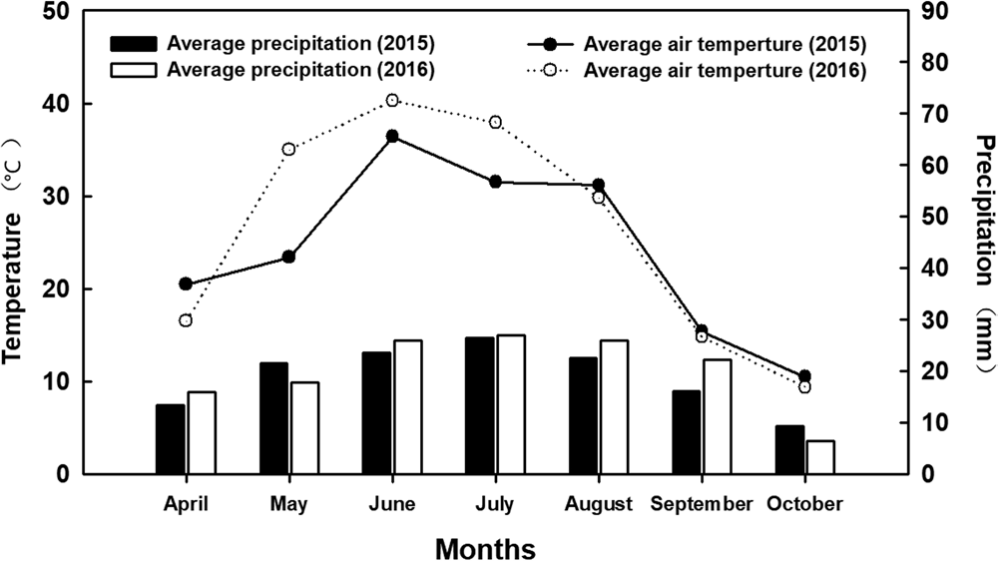
The monthly change in precipitation and air temperature in Shihezi during 2015–2016.

The experiment was conducted at a research station at Shihezi University, Xinjiang, northwestern China (45°19′N, 74°56′E) from April to October in 2015 and 2016. The maximum/minimum temperatures and mean precipitation for the growth season in both years are shown in Fig. 10. Cotton was grown in polyvinyl chloride (PVC) tubes (diameter: 30 cm; the tubes consisted of three stacked sections; each section was 40 cm high, and the total column height was 120 cm). The bottom of the tube was covered with a wire mesh fine enough to hold soil while allowing water to pass through. Clay loam soil collected from the field station was passed through a 2 mm sieve, packed in the PVC tubes in increments of 0.1 m to 1.2 m and then air dried. The bulk density of the soil was 1.43 g m^−3^. The soil composition was purple clay loam (pH: 7.6), with 1.45 g (total N) kg^−1^, 0.23 g (P_2_O_5_) kg^−1^, 149 g (total K) kg^−1^, and 12.5 g (organic matter) kg^−1^.

### Experimental design

The cotton cultivar Xinluzao 45 was selected for experimentation. A randomized complete block design was employed for four treatments with 4 replicates each. Twelve tubes per treatment were vertically buried in the field. The two water treatments were pre-plant irrigation [**W**_**80**_, watered with 0.28 m^3^ (80 ± 5% of field capacity) per tube before sowing] and no pre-plant irrigation (**W**_**0**_, no water was applied over the entire depth of the tube). Based on our previous study^48^ investigating the fertilizer production requirement of lint (i.e., more than 2,300 kg ha^−1^ settled fertilizer), basal fertilizer (2.76 g of N, 9.36 g of P_2_O_5_, 6.38 g of K_2_O per tube) was applied as two fertilization depth [i.e., surface application (**F**_**10**_, sufficient basal fertilizer in the 10 cm–20 cm layer before sowing and deep application (**F**_**30**_, sufficient basal fertilizer in the 30 cm–40 cm layer before sowing)]. Nitrogen was applied with the ratio of basal fertilizer to topdressing of 1:4. Phosphorus and potassium were supplemented as basal fertilization. Urea [CO(NH_2_)_2_, 46.0% N] at the rate of 13.8 g per tube was used for nitrogen, while 18 g of monopotassium phosphate [(KH_2_PO_4_) 52.0% P_2_O_5_ and 35.4% K_2_O] was used per tube for the application of aforementioned amounts of P_2_O_5_ and K_2_O.

Four seeds were sown at a depth of 3 cm in tube on April 25 and May 1 in the year 2014 and 2015. The seeds were spaced 10 cm apart in one direction and 20 cm apart in the other. Drip laterals (Beijing Lvyuan Inc., China) were installed on top of the tubes, and one emitter per tube was fixed at the center. To reduce evaporation, the top of the tube was covered with a polyethylene film. Each pot was drip-irrigated once every four days. The total amount of water supplied to the plants in the different treatments was 434 mm each year. Standard local pest control measures were adopted. At 39, 54, 69, 84 and 99 DAE, the leaf area and root morphology parameters were measured, and the root and shoot dry matter were sampled.

### The recorded parameters are described below

#### Root growth measurement

The root distribution was measured in the soil columns at 39, 54, 69, 84 and 99 DAE. Three tubes per treatment were carefully dug from the ground and cut into 20 cm (2015) or 10 cm (2016) segments (beginning at the top of each column). The segments were immersed in water for 1 h. Then, the roots from each soil layer were placed in a 0.5 mm sieve and rinsed with running water. Simultaneously, debris, weeds, and dead roots were sorted from the living roots by hand during washing based on the procedure described by Gwenzi *et al*.^49^. The living roots were placed in deionized water and stored in a refrigerator prior to analysis. Living roots from three of the columns were evenly spread in a plastic tray filled with deionized water and scanned using a flatbed scanner (300 dpi). The root images were analyzed using the WinRhizo image analysis software (Regent Instruments, Quebec, Canada), which was configured to measure the root length. After scanning, the roots were oven-dried at 80 °C for 48 h and weighed. The effective root length was the length of the root with a diameter less than 0.5 mm, which represented the primary part with a role in nutrient absorption^50^.

#### The leaf area (LA) and the ratio of the effective root length to the leaf area

The LA was measured using the LI-3000 leaf area meter (LI-COR Inc., NE, USA). The ratio of the effective root length to the leaf area represents coordination among the root, leaf area and soil environment according to Körner and Renhardt (1987)^51^.

#### The relative growth rate and the loading capacity of the root and leaf

The relative growth rate (RGR) for each plant was calculated using the following formula^52^: RGR = [lnW_2_ – lnW_1_]/T, where W_2_ and W_1_ were the effective root length or leaf area after a growth stage and a previous growth stage, respectively, and T was the days between the two growth stage measurements (i.e., 39 or 15 days).

The productive capacity of the leaf area and the effective root length were measured according to Wu (1992)^53^ as follows:

$${\rm{Loading}}\,{\rm{capacity}}={\rm{total}}\,{\rm{dry}}\,\mathrm{matter}/\mathrm{leaf}\,{\rm{area}}\,{\rm{or}}\,{\rm{effective}}\,{\rm{root}}\,{\rm{length}}.$$

$${\rm{Loading}}\,{\rm{boll}}\,{\rm{capacity}}={\rm{reproductive}}\,{\rm{organ}}\,{\rm{dry}}\,\mathrm{matter}/\mathrm{leaf}\,{\rm{area}}\,{\rm{or}}\,{\rm{the}}\,{\rm{effective}}\,{\rm{root}}\,{\rm{length}}.$$

#### Biomass production, partitioning and the root/shoot ratio

A total of 12 cotton plants (i.e., three PVC tubes) were selected from each treatment and cut at the cotyledonary node. The plants were separated into leaves, stems, buds, flowers, bolls and roots. The dry mass of the plant samples oven-dried at 80 °C was measured. The partitioning rate was the ratio of the organ (i.e., leaves, stems, buds, flowers, bolls or roots) dry matter to the total dry matter.

### Statistical analysis

Analysis of variance (ANOVA), correlation analysis and path analysis were performed using the SPSS software version 16.0 (SPSS Inc., Chicago, IL, USA). Differences between treatments were considered significant at *P* < 0.05 according to least significant difference (LSD) test. The figures were plotted using the Sigma Plot version 10.0 software (Systat Software Inc., San Jose, CA, USA), and the data are presented as the mean ± SD.
